# Apport de la qPCR dans le diagnostic des infections cervicovaginales à l'Hôpital Principal de Dakar, Sénégal

**DOI:** 10.48327/mtsi.v4i1.2024.298

**Published:** 2024-02-05

**Authors:** Aminata Sarif DIALLO, Mor NGOM, Sokhna Moumy MBACKÉ DAFFE, Hubert BASSÈNE, Masse SAMBOU, Yakhya DIEYE, Bécaye FALL, Cheikh SOKHNA

**Affiliations:** 1 UMR 257 Vecteurs-Infections Tropicales et Méditerranéennes (VITROME), Campus international IRD-UCAD, Dakar, Sénégal; 2 Département PCL/Sciences, Institut supérieur des sciences de l’éducation de Guinée, Sénégal; 3 Service de biologie, Département formation des professeurs de collège et lycée Sciences fondamentales et des animateurs pédagogiques de l'enseignement secondaire (DFPCL-SF/APES), Institut supérieur des sciences de l’éducation de Guinée, Fédération des laboratoires, Hôpital Principal de Dakar, Sénégal; 4 Pôle de microbiologie, Institut Pasteur de Dakar, Sénégal Auteur

**Keywords:** Infections cervico-vaginales, Cytobactériologie, qPCR, Hôpital, Dakar, Sénégal, Afrique subsaharienne, Cervico-vaginal infections, Cytobacteriology, qPCR, Hospital, Dakar, Senegal, Sub-Saharan Africa

## Abstract

**Objectif:**

Déterminer l’étiologie des infections cervico-vaginales par la cytobactériologie et l'efficacité de la qPCR pour le diagnostic des souches sensibles telles que *Streptococcus agalactiae, Borrelia crocidurae, Chlamydia trachomatis, Neisseria gonorrhoeae* et *Treponema pallidum.*

**Méthodologie:**

Étude prospective transversale effectuée entre janvier et septembre 2021 chez 346 femmes reçues à l'Hôpital Principal de Dakar pour une infection cervico-vaginale. Des analyses cytobactériologiques et moléculaires ont été réalisées.

**Résultats:**

Les déséquilibres de la flore vaginale ont été prédominants, avec un taux de 72,3 %. La proportion des flores vaginales de type IV a été de 46,5 %. Sur les 199 germes isolés, *Candida albicans* (25,1 %), *Ureaplasma urealyticum* (17,6 %), S. *agalactiae* (7,8 %), *Gardnerella vaginalis* (6,6 %) et *Candida non-albicans* (5,5 %) ont été les principaux pathogènes responsables des infections cervico-vaginales chez les patientes. Chez les femmes concernées par une recherche de mycoplasmes, U. *urealyticum* a été identifié chez 43,3 % des patientes. Chez celles qui étaient concernées par une recherche de C. *trachomatis,* la proportion de femmes infectées a été faible (4 %). Les différentes méthodes ayant montré de faibles prévalences de C. *trachomatis* et de N. *gonorrhoeae,* les comparaisons Test RapidChlamydia/qPCR pour C. *trachomatis* et culture/qPCR pour N. *gonorrhoeae* n'ont pas été possibles. Par contre, pour *S. agalactiae,* la qPCR a été plus avantageuse que la culture. Les isolats de S. *agalactiae* et d'entérobactéries présentaient successivement une résistance élevée à l'acide nalidixique et à l'ampicilline.

**Conclusion:**

Les méthodes appliquées ont permis d'identifier les pathogènes qui sont à l'origine des infections cervico-vaginales. Les résultats suggèrent que la qPCR peut être une alternative au moins pour le diagnostic de S. *agalactiae.* Cependant la culture reste indispensable pour étudier la sensibilité aux antibiotiques. Dans un souci d'amélioration de la prise en charge des patientes, les techniques moléculaires doivent être intégrées dans la panoplie des tests à l'Hôpital Principal de Dakar (HPD).

## Abstract (en)

**Objective:**

To determine the etiology of cervico-vaginal infections by cytobacteriology and the efficacy of qPCR for the diagnosis of sensitive strains such as *Streptococcus agalactiae, Borrelia crocidurae, Chlamydia trachomatis, Neisseria gonorrhoeae* and *Treponema pallidum.*

**Methodology:**

This prospective cross-sectional study was performed between January and September 2021 in 346 women who were examined for cervico-vaginal infection at the Hôpital Principal de Dakar (HPD). Cytobacteriological (direct examination, agar culture) and molecular analyses were performed.

**Results:**

Vaginal flora imbalances predominated, with a rate of 72.3%. The proportion of type IV vaginal flora was 46.5%. Of the 199 germs isolated, *Candida albicans* (25.1%), *Ureaplasma urealyticum* (17.6%), *S. agalactiae* (7.8%), *Gardnerella vaginalis* (6.6%) and nonalbicans *Candida* (5.5%) were the main pathogens responsible for cervico-vaginal infections in patients. Among women tested for mycoplasma, *U. urealyticum* was identified in 43.3% of patients. Among those tested for *C. trachomatis,* the proportion of infected women was low (4%). The prevalence of *C. albicans* was higher in pregnant women (38.3%) than in nonpregnant women (19.2%). *S. agalactiae* strains showed high resistance to certain beta-lactam antibiotics (pristinamycin 100%, gentamycin 100%, ampicillin 92.5% and cefalotin 85.2%) and to a glycopeptide antibiotic (vancomycin 100%). The *Staphylococcus aureus* strain had good sensitivity to antibiotics except gentamycin (100%) and kanamycin (100%). The enterobacteria tested were all sensitive to phenicols, carbapenems, cephalosporins and aminoglycosides. However, *E. coli* showed high resistance to tetracycline. The different methods showed low prevalences of *C. trachomatis* and *N. gonorrhoeae,* so comparisons Test RapidChlamydia/qPCR for *C. trachomatis* and culture/qPCR for N. *gonorrhoeae* were not possible. For *S. agalactiae,* on the other hand, qPCR was more advantageous than culture. The χ^2^ test showed a significant difference (Yates χ^2^ = 33.77 and p = 1^-7^) for the diagnosis of *S. agalactiae. S. agalactiae* qPCR had a sensitivity of 40.7%, a specificity of 94%, and positive and negative predictive values of 36.7% and 94.9% respectively, as well as a kappa = 0.33.

**Conclusion:**

The methods applied enabled us to identify the pathogens that cause cervicovaginal infections. The results suggest that qPCR may be an alternative, at least for the diagnosis of *S. agalactiae.* However, culture remains indispensable for studying antibiotic sensitivity. In order to improve patient care, molecular techniques need to be integrated into the HPD testing toolbox. To broaden the repertoire of pathogens to be diagnosed by qPCR, targeted comparison studies will be needed to increase the probability of encountering infected individuals.

## Introduction

Les infections de l'appareil reproducteur (IAR), y compris les infections sexuellement transmissibles (IST), constituent un problème majeur de santé publique dans le monde, en particulier dans les pays en développement [1]. Les infections vaginales (bactériennes, fongiques et parasitaires) touchent les femmes du monde entier et peuvent être d'origine exogène ou endogène. Elles sont le reflet d'une altération du microbiote vaginal dont l’équilibre est modifié par une prolifération de bactéries pathogènes. Les prévalences les plus importantes sont rencontrées chez les femmes en âge de procréer. Au Sénégal, une étude rétrospective menée à l'Hôpital militaire de Ouakam, Dakar, a montré une prévalence importante de *Candida albicans* dans des écouvillons vaginaux de femmes [11]. En revanche, Barry *et al.* ont trouvé une prédominance des vaginoses bactériennes (39,5 %) par rapport aux candidoses vaginales (29 %) [1]. Des pathogènes tels que *Chlamydia trachomatis, Neisseria gonorrhoeae* et *Trichomonas vaginalis* sont à l'origine de la majorité des IST traitables dans le monde [7]. Les IST non traitées telles que celles dues à *C. trachomatis, N. gonorrhoeae, T. vaginalis* et *Mycoplasma genitalium* peuvent augmenter le risque d'acquisition du virus de l'immunodéficience humaine (VIH), de maladies inflammatoires pelviennes ou de naissances prématurées [6]. *Streptococcus agalactiae* (streptocoque du groupe B) est un membre du microbiote commensal des voies intestinales et génito-urinaires humaines et est également un agent pathogène important dans la vaginite aérobie et d'autres infections humaines [4]. Bien que l'infection à *Listeria monocytogenes* soit parfois paucisymptomatique chez les femmes, un avortement spontané peut survenir dans environ 30 % des cas [5]. Au Sénégal, une étude menée dans les sites de Dielmo, Ndiop et Niakhar a montré deux cas de L. *monocytogenes* (5 %) parmi 43 femmes ayant eu des fausses couches [3]. Ces infections prennent une part importante dans les motifs de consultation en gynécologie. Les complications sont nombreuses dont le cancer du col de l'utérus, les avortements spontanés ou les infections néonatales. À ce stade, le coût de la prise en charge médicale et du traitement peut être très élevé et ainsi constituer un facteur limitant dans la lutte contre ces infections.

Dans beaucoup de structures sanitaires du Sénégal, le diagnostic bactériologique se fait en grande partie par un examen cytobactériologique classique. Il consiste à examiner directement les cellules et les bactéries au microscope optique (état frais et après coloration), puis à mettre les échantillons en culture et enfin analyser la sensibilité des souches isolées aux antibiotiques. Or, certaines bactéries d'intérêt médical majeur sont fragiles ou difficilement cultivables *(C. trachomatis, N. gonorrhoeae,* mycoplasmes). Les données sur le profil épidémiologique des infections cervico-vaginales restent limitées au Sénégal. Par ailleurs, l'accès pour tous au diagnostic de laboratoire est un défi pour les autorités sanitaires. Les prix appliqués pour un diagnostic cytobactériologique de prélèvements vaginaux à l'Hôpital Principal de Dakar (HPD) sont de 25 000 FCFA (38,1 €), et de 60 000 FCFA (91,5 €) lorsqu'il est accompagné d'une recherche de *C. trachomatis.* Malgré ces contraintes financières, peu d’études se sont intéressées à l'apport des techniques moléculaires dans le diagnostic d'infections cervico-vaginales concomitamment avec la bactériologie classique. L'objectif de notre étude était de déterminer l’étiologie des infections cervico-vaginales, d’évaluer et de comparer les méthodes de diagnostic cytobactériologique et moléculaire appliquées pour l'identification des pathogènes en cause.

## Matériels et méthodes

### Population de l’étude

Il s'agissait d'une étude observationnelle prospective réalisée du 4 janvier au 13 septembre 2021. L’étude concernait les femmes reçues au Centre autonome de prélèvements externes pour un prélèvement vaginal et les patientes suivies en interne à l'Hôpital Principal de Dakar (HPD) présentant des signes d'infection cervico-vaginale. Celles dont les questionnaires d'enquête étaient incomplets ont été exclues de l’étude.

### Considérations éthiques

Cette étude a été réalisée avec le consentement libre et éclairé des patientes qui ont donné leur autorisation pour l'utilisation des prélèvements à des fins scientifiques. Des fiches d'information et de consentement ont été élaborées à cet effet. Ces documents renseignaient sur les objectifs, les risques, les avantages et les inconvénients de l’étude.

### Collecte des échantillons biologiques

Des écouvillons secs (Deltalab, S.L. PI. La Vermeda 1 08191 Rubi-BCN, Espagne) ont été utilisés pour l'examen cytobactériologique et des transwabs (Corsham, Wiltshire, SN13 9RT, Royaume-Uni, Réf. MW1766) pour l'analyse moléculaire. Tous les prélèvements ont été réalisés en insérant les écouvillons successivement dans le canal vaginal jusqu’à la muqueuse du col de l'utérus et dans le canal endocervical. Les écouvillons secs ont été immédiatement analysés au laboratoire de l'HPD et les transwabs ont été aliquotés puis conservés à -80 °C jusqu'au moment de les analyser.

### Diagnostic cytobactériologique

Une goutte de la suspension des écouvillons secs dans le bouillon acide aminé (AA) a été placée entre lame et lamelle, puis observée au microscope à l'objectif 40X. Trois milieux ont été ensemencés : Sabouraud, gélose au sang frais et gélose au sang frais supplémentée d'acide nalidixique et de colistine (ANC). puis incubés pendant 24 heures à 37 °C, en aérobie. Les géloses au sang cuit (supplémenté de polyvitamines) ensemencées dans la salle de prélèvement pour la recherche de *N. gonorrhoeae* étaient incubées en atmosphère de 5 % de CO_2_. Lexsudat de l’écouvillonnage du canal vaginal a été étalé sur une lame, fixé à la flamme puis coloré au Gram. Les frottis ont été examinés au microscope optique à immersion dans l'huile (100X) pour observer la présence ou l'absence d'un déséquilibre de la flore vaginale. Les déséquilibres sont caractérisés par la disparition de la flore normale au détriment d'une flore aérobie *(Escherichia coli, S. agalactiae, Enterococcus faecalis, Staphylococcus aureus..)* ou par d'autres microorganismes dits commensaux *(Ureaplasma urealyticum).* La méthode décrite par Nugent en 1991 [9] a été utilisée pour analyser les déséquilibres de la flore vaginale. Elle consiste à évaluer de façon semi-quantitative l’équilibre de la flore vaginale en fonction de trois morphotypes bactériens : *Lactobacillus* spp, *Gardnerella vaginalis, Mobiluncus* spp. Un score supérieur à 7 définit la vaginose bactérienne.

La sensibilité aux antibiotiques a été déterminée sur les colonies suspectes isolées après purification. Nous avons utilisé des cartes antibiogrammes prêtes à l'emploi de type Vitek* 2 de Biomérieux, selon les instructions du fabricant. Les cartes suivantes ont été utilisées : AST 667 pour le *S. agalactiae,* AST 234 pour les entérobactéries et AST 580 pour le *S. aureus. C*. *trachomatis* a été mis en évidence par le test immunochromatographique « Test RapidChlamydia, RightSign » (TRC) (Hangzhou Biotest Co, Ltd, Réf. WCHL-C71). La recherche de C. *trachomatis* et des mycoplasmes dans notre étude était conditionnée à une prescription médicale. Le diagnostic d'U. *urealyticum* et de *Mycoplasma hominis* a été réalisé grâce au test « Nadal *Mycoplasma* » (Nal Von Minden GmbH, Réf. 1770001, Lot Q22L2004) qui permet à la fois d'identifier les mycoplasmes et de déterminer leur sensibilité aux antibiotiques. Les deux tests ont été réalisés conformément aux instructions des fabricants.

### Analyse moléculaire

L'ADN a été extrait des écouvillons dans un volume de 200 pl à l'aide de la méthode au cetyltrimethylammonium bromide (CTAB) 2 % [10]. Les échantillons ont été conservés entre 2 et 8 °C jusqu'au moment de leur utilisation. Cinq bactéries *(S. agalactiae, Borrelia crocidurae, C. trachomatis, N. gonorrhoeae* et *Treponema pallidum)* ont été recherchées par la PCR à temps réel. La qPCR a été réalisée avec un thermocycleur de type CFX Connect (788BR09570, Bio-Rad, Singapour) en mélangeant 5 pl d'ADN et 15 pl de mix PCR composés de 10 pl de Probe Master (LightCycler 480, Roche Diagnostics GmbH, Allemagne), 3,5 pl de H_2_O, 0,5 pl pour chaque amorce et sonde spécifiques (Tableau I). Les conditions de l'amplification étaient les suivantes : un cycle de dénaturation initiale à 95 °C (10 minutes), suivi de 40 cycles de dénaturation à 95 °C (10 secondes) et d'hybridation à 60 °C (30 secondes). Un échantillon a été considéré comme positif lorsque la valeur du Ct était < 35 cycles. Les séquences des amorces et de la sonde pour la détection de *S. agalactiae* ont été fournies par l'IHU Marseille.

**Tableau I:** Liste et séquences des amorces et des sondes utilisées pour la qPCR, Hôpital Principal de Dakar, 2021 List and sequences of primers and probes used for qPCR, Hôpital Principal de Dakar, 2021

Espèces	Gènes	Séquences des amorces (F et R) et sondes (P)	Référence
*Chlamydia trachomatis*	C tracho-1	F : 5’-AGCT CCCAAAGCAACCAGAR-3’	[2]
		R : 5’-BTGTCGCTGCGTTGGTTTTA-3’	
		P : 6-FAM- CAACAGCACCACCAGCAGCTGC	
*Borrelia crocidurae*	16S	F : 5’-AGCCT T TAAAGCT T CGCTTGTAG-3’	[8]
		R : 5’-GCCTCCCGTAGGAGTCTGG-3’	
		P : 6FAM- CCGGCCTGAGAGGGTGAACGG	
*Neisseria gonorrhoeae*	N gono-2	F : 5’-CATCAGCGCAATCCCTATGA-3’	[2]
		R : 5’-TGGTTGTCCTCAACCGTGTC-3’	
		P : 6FAM- CCACGGCGCTTCCTGATGGG	
*Streptococcus agalactiae*	Saga Cfb	F : 5’-ATTGCGTGCCAACCCTGAG-3’	
		R : 5’-AAGGCT T CTACACGACTACCA-3’	
		P : 6FAM- CAGTTTATGATTTGAATTCT -MGB	
*Treponema pallidum*	Syph T	F : 5’-GT CGACACT GAAAAGGAGT GCA-3’	[2]
		R : 5’-GTCAGCGTCTCATCATTCCAAAG-3’	
		P : 6FAM- TGCTGTGCAGGATCCGGCQTQTGTC -TAMRA	

### Analyses statistiques

Les données ont été saisies sur Excel (Microsoft Office 2010). Les analyses statistiques ont été effectuées sur Epi Info 7.1.3.3 (CDC, Atlanta, États-Unis). Des statistiques descriptives ont été appliquées pour déterminer la distribution des caractéristiques sociodémographiques et cliniques. Le test de Chi d'indépendance a été utilisé pour comparer les résultats des deux méthodes. Nous avons considéré les différences comme significatives pour une valeur de p < 0,05. La sensibilité, la spécificité, les valeurs prédictives positives et négatives (VPP et VPN) ont été calculées pour la qPCR en la comparant aux méthodes usuelles de culture utilisées pour le diagnostic des pathogènes.

## Résultats

### Caractéristiques des patientes

Trois cent quarante-six (346) patientes ont été incluses dans l’étude, âgées de 18 à 70 ans, avec un âge moyen de 35 ans (intervalle de confiance à 95 % de ± 0,98) et 91 % (315/346) étaient âgées de 20 à 49 ans. Les femmes mariées et les nullipares représentaient 93,4 % (323/346) et 36,1 % (125/346) respectivement. Les femmes suivies en interne et celles qui ont bénéficié d'un examen bactériologique des sécrétions vaginales étaient 54,9 % (190/346) et 86,4 % (299/346). Concernant les symptômes cliniques, 54,6 % (189/346) avaient des douleurs abdomino-pelviennes et plus de 99 % (344/346) avaient des leucorrhées. La majorité des demandes était des bilans infectieux (37 %, 128/346) suivis du bilan prénatal (29,2 %, 101/346). La recherche de C. *trachomatis* concernait 43,4 % de ces patientes (150/346) et les mycoplasmes ont été recherchés chez 40,8 % (141/346). Ces recherches ont été suivies par des prescriptions médicales. Les caractéristiques sociodémographiques et cliniques des patientes sont résumées dans le Tableau II.

**Tableau II:** Caractéristiques sociodémographiques et cliniques des 346 patientes, Hôpital Principal de Dakar, 2021 Sociodemographic and clinical characteristics of the 346 patients, Hôpital Principal de Dakar, 2021

Variables	Nombre	Pourcentage
**Tranches d’âge**		
[20-29]	99	28,6 %
[30-39]	149	43,1 %
[40-49]	67	19,4 %
> 49	27	7,8 %
< 20	4	1,2 %
**Situation matrimoniale**		
mariées	323	93,4 %
célibataires	10	2,9 %
divorcées	7	2 %
veuves	6	1,7 %
**Parturité**		
nullipares	125	36,1 %
paucipares	102	29,5 %
primipares	72	20,8 %
multipares	47	13,6 %
**Grossesse**		
enceintes	107	30,1 %
non enceintes	239	69,1 %
**Origine des participantes**		
internes (suivies à HPD)	190	54,9 %
externes	148	42,8 %
ND	8	2,3 %
**Type d'examen**		
examen bactériologique (EB)	346	100 %
**Contraception**		
utilisatrices	32	9,2 %
non utilisatrices	314	90,8 %
**Diabète**		
diabétiques	15	4,3 %
non diabétiques	331	95,7 %
**Douleurs abdomino-pelviennes**				
oui	189	54,6 %
non	157	45,4 %
**Abondance des leucorrhées**		
très abondantes	39	11,3 %
abondantes	131	37,9 %
moyennes	174	50,3 %
absentes	2	0,6 %
**Dysurie**		
présence	49	14,2 %
absence	297	85,8 %
**Inflammation du col**		
présence	57	16,5 %
absence	289	83,5 %
**Saignement du col au contact**		
présence	55	15,9 %
absence	291	84,1 %
Flore vaginale*		
type II	94	27,2 %
type III	91	26,3 %
type IV**	161	46,5 %
**Motif de consultation**		
bilan infectieux	128	37 %
bilan prénatal	101	29,2 %
infertilité	55	15,9 %
contrôle	14	4 %
métrorragies	13	3,8 %
bilan post-avortement	12	3,5 %
aménorrhée	12	3,5 %
MAP	5	1,4 %
bilan post-partum	3	0,9 %
bilan préopératoire	2	0,6 %
décollement du placenta	1	0,3 %
**Infections isolées**		
cervicite	7	2 %
déséquilibre (contexte inflammatoire)	155	44,8 %
déséquilibre (sans contexte inflammatoire)	95	27,5 %
vaginite	78	22,5 %

* Type II : lactobacillus majoritaires associés à peu de bactéries; Type III : minorité de lactobacillus; Type IV : absence de lactobacillus.

** Dans notre étude, la vaginose bactérienne est définie par la flore de type IV

MAP : Menace d'accouchement prématuré

ND : non définie

### Diagnostic cytobactériologique

La plupart des patientes avaient une flore vaginale de type IV (46,5 %, 161/346). Les déséquilibres de la flore vaginale étaient les plus fréquents (72,3 %, 250/346), suivis des vaginites (22,5 %, 78/346). Parmi les déséquilibres et les vaginites associés, 71 % (233/328) étaient associés à une inflammation et 29 % (95/328) sans inflammation. Le portage normal de C. *albicans* et les cervicites concernaient 3,2 % (11/346) et 2 % (7/346) des femmes. Sur les 346 écouvillons, 57,5 % (199/346) étaient au moins positifs à un microorganisme (Tableau III). C. *albicans* représentait 25,1 % (87/346) des germes identifiés, suivi *dU. urealyticum* (17,6 %, 61/346), de *S. agalactiae* (7,8 %, 27/346), de *G. vaginalis* (6,6 %, 23/346) et de C. *non-albicans* (5,5 %, 19/346). *U. urealyticum* a été le pathogène cervical le plus retrouvé (43,3 %, 61/141) comparé à C. *trachomatis* (4 %, 6/150).

En fonction du type de flore, les résultats ont montré un taux plus élevé des germes chez les patientes avec une flore de type IV (64 %, 103/161) que chez celles à flore de type III (58,2 %, 53/91) ou de type II (46 %, 43/94). La différence entre les taux globaux de positivité était significative (χ^2^ = *7,35; p* = 0,017) (Tableau IV).

**Tableau III:** Prévalence des germes isolés chez les 346 patientes, Hôpital Principal de Dakar, 2021 Prevalence of germs isolated among 346 patients, Hôpital Principal de Dakar, 2021

Types d'infections	Germes	Positifs	Pourcentage
Infections simples	*C. albicans*	67	19,4 %
	*U. urealyticum*	37	10,7 %
	*G. vaginalis*	16	4,6 %
	*S. agalactiae*	16	4,6 %
	*C. non-albicans*	13	3,8 %
	*C. trachomatis*	5	1,5 %
	*E. coli*	2	0,6 %
	*E. feacalis*	2	0,6 %
	*T vaginalis*	2	0,6 %
	*N. gonorrhoeae*	1	0,3 %
	*S. aureus*	1	0,3 %
Infections mixtes	*C. albicans / U. urealyticum*	8	2,3 %
	*U. urealyticum / S. agalactiae*	6	1,7 %
	*C. albicans / G. vaginalis*	2	0,6 %
	*C. albicans / G. vaginalis / U. urealyticum*	2	0,6 %
	*C. albicans / S. agalactiae*	2	0,6 %
	*C. non-albicans / S. agalactiae*	2	0,6 %
	*G. vaginalis / U. urealyticum*	2	0,6 %
	*C. albicans / Citrobacter koseri*	1	0,3 %
	*C. albicans / E. coli*	1	0,3 %
	*C. albicans / Klebsiella pneumoniae*	1	0,3 %
	*C. albicans / E. feacalis*	1	0,3 %
	*C. albicans / U. urealyticum / M. hominis*	1	0,3 %
	*C. albicans / U. urealyticum / S. agalactiae*	1	0,3 %
	*C. non-albicans / E. coli*	1	0,3 %
	*C. non-albicans / G. vaginalis / E. coli*	1	0,3 %
	*C. non-albicans / E. feacalis*	1	0,3 %
	*C. non-albicans / U. urealyticum*	1	0,3 %
	*C. trachomatis / U. urealyticum*	1	0,3 %
	*U. urealyticum / E. coli*	1	0,3 %
	*U. urealyticum / M. hominis*	1	0,3 %
Total		199	57,5 %

**Tableau IV:** Distribution des germes en relation avec la flore vaginale des 346 patientes, Hôpital Principal de Dakar, 2021 Distribution of germs in relation to the vaginal flora of 346 patients, Hôpital Principal de Dakar, 2021

Germes isolés	Type de flore vaginale			Total
	Type II	Type III	Type IV	
*C. albicans*	20	18	29	67
*C. albicans / Citrobacter koseri*	0	1	0	1
*C. albicans / Escherichia coli*	0	0	1	1
*C. albicans / G. vaginalis*	0	0	2	2
*C. albicans / G. vaginalis / U. urealyticum*	0	0	2	2
*C. albicans / K. pneumoniae*	0	0	1	1
*C. albicans / S. agalactiae*	0	1	1	2
*C. albicans / E. feacalis*	0	0	1	1
*C. albicans / U. urealyticum*	3	3	2	8
*C. albicans / U. urealyticum / M. hominis*	0	0	1	1
*C. albicans / U. urealyticum / S. agalactiae*	0	0	1	1
*C.* non*-albicans*	5	3	5	13
*C.* non*-albicans / E. coli*	0	0	1	1
*C.* non*-albicans / G. vaginalis / E. coli*	0	0	1	1
*C.* non*-albicans / S. agalactiae*	0	1	1	2
*C.* non*-albicans / E. feacalis*	0	0	1	1
*C.* non*-albicans / U. urealyticum*	0	0	1	1
*C. trachomatis*	3	2	0	5
*C. trachomatis / U. urealyticum*	0	1	0	1
*E. coli*	0	1	1	2
*G. vaginalis*	1	0	15	16
*G. vaginalis / U. urealyticum*	0	0	2	2
*N. gonorrhoeae*	0	0	1	1
*S. aureus*	0	1	0	1
*S. agalactiae*	2	5	9	16
*E. feacalis*	0	1	1	2
*T. vaginalis*	0	0	2	2
*U. urealyticum*	9	13	15	37
*U. urealyticum / E coli*	0	0	1	1
*U. urealyticum / M. hominis*	0	0	1	1
*U. urealyticum / S. agalactiae*	0	2	4	6
**Total positifs**	**43**	**53**	**103**	**199**

La prévalence de *C. albicans* était significativement plus élevée chez les femmes enceintes (38,3 %, 41/107) contre 19,4 % (46/237) chez celles qui ne l’étaient pas (χ^2^ *= 13,28*; *p* = 0,0002).

### Sensibilité aux antibiotiques

Les 27 isolats de *S. agalactiae* présentaient des sensibilités élevées aux bêta-lactamines (pristinamycine 100 %, ampicilline 92,6 % et céfalotine 85,2 %) et à un antibiotique de la famille des glycopeptides (vancomycine 100 %). Des résistances élevées à la gentamycine (100 %) et à l'acide nalidixique (81 %) ont été notées. La souche de *S. aureus* avait une bonne sensibilité aux antibiotiques testés, excepté à la gentamycine (100 %) et à la kanamycine (100 %). Les entérobactéries testées ont toutes été sensibles aux phénicolés, carbapénèmes, céphalosporines et aminosides. *E. coli* a été sensible à la doxycycline tandis qu'une résistance importante à la tétracycline a été notée (67 %). Concernant toujours les entérobactéries, les sensibilités aux bêta-lactamines ont été diverses (Tableau V).

**Tableau V:** Prévalence des germes isolés chez les 346 patientes, Hôpital Principal de Dakar, 2021 Prevalence of germs isolated among 346 patients, Hôpital Principal de Dakar, 2021

Antibiotiques	*S. agalactiae* (N=27)			*E. feacalis* (N=3)			*S. aureus* (N=1)		*E. coli* (N=6)			*C. koseri* (N=1)			*K. pneumoniae* (N=1)		
	S %	R %	I %	S %	R %	I %	S %	R %	S %	R %	I %	S %	R %	I %	S %	R %	I %
Pénicilline G	70,4(19)	18,5(5)	11,1(3)	0	66,7(2)	33,3(1)	100	-	-	-	-	-	-	-	-	-	-
Ampicilline	92,6(25)	3,7(1)	3,7(1)	100(3)	0	0	-	-	16,7(1)	50(3)	33,3(2)	0	100	0	0	100	0
Céfalotine	85,2(23)	7,4(2)	3,7(1)	0	66,77(2)	33,3(1)	-	-	83,3(5)	0	16,7(1)	100	0	0	100	0	0
Vancomycine	100(27)	0	0	100(3)	0	0	100	-	-	-	-	-	-	-	-	-	-
Gentamycine	0	100(27)	0	0	100(3)	-	0	100	100(6)	0	0	100	0	0	100	0	0
Kanamycine	0	100(27)	0	0	100(3)	0	0	100	-	-	-	-	-	-	-	-	-
Érythromycine	77,8(21)	18,5(5)	0	0	100	0	100	-	83,3(5)	16,7(1)	0	-	-	-	-	-	-
Lincomycine	81,5(22)	18,5(5)	0	0	100	0	100	-	-	-	-	-	-	-	-	-	-
Pristinamycine	100(27)	0	0	33,3(1)	66,7(2)	0	100	-	100(6)	0	0	-	-	-	-	-	-
Acide nalidixique	19(5)	81,5(22)	0	0	100(3)	0	-	-	66,7(4)	16,7(1)	0	100	0	0	100	0	0
Rifampicine	81,5(22)	11,1(3)	7,4(2)	0	100(3)	0	100	-	-	-	-	-	-	-	-	-	-
Tétracycline	33,3(9)	59,3(16)	3,7(1)	0	100(3)	0	-	-	33,3(2)	66,7(4)	0	-	-	-	-	-	-
Doxycycline	77,8(21)	22,2(6)	0	66,7(2)	33,3(1)	0	100	-	100(6)	0	0	-	-	-	-	-	-
Cotrimoxazole	40,7(11)	51,9(14)	0	66,7(2)	33,3(1)	0	100	-	33,3(2)	50(3)	0	100	0	0	0	100	0
Chloramphénicol	92,6(25)	7,4(2)	0	3,33(1)	33,3(1)	0	100	-	100(6)	0	0	100	0	0	100	0	0
Oxacilline	-	-	-	-	-	-	100	-	-	-	-	-	-	-	-	-	-
Amox + Acide clavul	-	-	-	-	-	-	-	-	100	0	0	100	0	0	100	0	0
Ticarcilline	-	-	-	-	-	-	-	-	50(3)	3,33(2)	16,7(1)	0	100	0	0	100	0
Imipénème	-	-	-	-	-	-	-	-	100(6)	0	0	100	0	0	100	0	0
Céfoxitine	-	-	-	-	-	-	-	-	100(6)	0	0	100	0	0	100	0	0
Ceftriaxone	-	-	-	-	-	-	-	-	100(6)	0	0	100	0	0	100	0	0
Céfépime	-	-	-	-	-	-	-	-	100(6)	0	0	100	0	0	100	0	0
Nétilmicine	-	-	-	-	-	-	-	-	83,3(5)	0	0	100	0	0	100	0	0
Colistine	-	-	-	-	-	-	-	-	66,7(4)	0	0	100	0	0	100	0	0

-: non testé; Amox + Acide clavul : amoxicilline + acide clavulanique; S % : taux de sensibilité; R % : taux de résistance; I % : taux de sensibilité intermédiaire.

### Facteurs de risque d'acquisition de certaines infections vaginales

Dans l'analyse bivariée, aucune corrélation n'a été observée entre la grossesse et la présence d'U. *urealyticum, S. agalactiae.* Le résultat en fonction de la grossesse est déjà présenté plus haut.

### Diagnostic moléculaire

Sur les 346 écouvillons analysés, 34 étaient positifs à la PCR. Les pathogènes identifiés ont été : S. *agalactiae* (8,4 %, 29/346), *N. gonorrhoeae* (0,9 %, 3/346), C. *trachomatis* (0,3 %, 1/346) et une co-infection *S. agalactiae* / C. *trachomatis* (0,3 % 1/346). Les autres pathogènes ciblés ont été absents.

### Analyse comparée des résultats de la cytobactériologie et de la qPCR

La bactérie C. *trachomatis* a été recherchée chez 150 patientes. Pour chaque échantillon, une qPCR et un Test RapidChlamydia (TRC) ont été réalisés. Les résultats ont montré une prévalence faible avec 4 % (6/150) par le TRC et 1,3 % (2/150) par la qPCR. Aucun échantillon n'a été positif à la fois par le TRC et la qPCR. *N. gonorrhoeae* a été recherchée chez 346 patientes. Sa prévalence en culture était également faible (0,3 %, 1/346) contre 0,9 % (3/346) par qPCR. Pour *S. agalactiae*, 11 échantillons ont été à la fois positifs à la qPCR et à la culture (Tableau VI). La qPCR utilisée pour la détection de *S. agalactiae* a identifié 19 échantillons positifs qui ont été négatifs en culture. Par contre 16 échantillons ont été positifs en culture et négatifs en qPCR. Le test de χ^2^ d'indépendance effectué montre une différence statistiquement significative (χ^2^ de Yates = 33,77 et *p =* 1^-7^) pour *S. agalactiae.* Considérant la culture comme gold standard, la qPCR *S. agalactiae* avait une sensibilité de 40,7 %, une spécificité de 94 %, des valeurs prédictives positive et négative de 36,7 % et 94,9 % respectivement. La concordance entre les deux méthodes de détection de *S. agalactiae* était faible : nous avons obtenu un kappa = 0,33.

**Tableau VI:** Comparaison entre le test qPCR et la culture/Test RapidChlamydia pour la détection des trois pathogènes, Hôpital Principal de Dakar, 2021 Comparison between the qPCR test and the culture/RapidChlamydia test for the detection of the three pathogens, Hôpital Principal de Dakar, 2021

qPCR / TRC (ou culture)	+/+	+/-	-/+	-/-	Total	Kappa de Cohen	χ^2^ (p)
*C. trachomatis*	0	2	6	142	150	-0,02	0,015 (0,7)
*N. gonorrhoeae*	0	3	1	342	346	-0,004	0,023 (0,9)
*S. agalactiae*	11	19	16	300	346	0,331	33,77 (1^-7^)

+/+ = échantillons positifs à la qPCR et au TRC (ou culture); +/- = échantillons positifs à la qPCR, négatifs au TRC (ou culture); -/+ = échantillons négatifs à la qPCR, positifs au TRC (ou culture); -/- = échantillons négatifs à la qPCR et au TRC (ou culture) Trois souches de *S. agalactiae* nont pas été testées à la céfalotine, érythromycine, tétracycline; deux autres souches nont pas été testées au cotrimoxazole. Une souche d’*E*. *coli* n'a pas été testée à la nétilmicine, à l'acide nalidixique et au cotrimoxazole.

## Discussion

Cette étude visait à déterminer l’étiologie des infections cervico-vaginales chez les femmes reçues au Centre autonome de prélèvements externes, par les méthodes cytobactériologiques et moléculaires. Elle visait également à comparer les méthodes de diagnostic cytobactériologique et moléculaire appliquées à la détection de certains pathogènes dont la culture peut être difficile du fait de leurs sensibilités. L'examen bactériologique était le motif de prélèvement le plus partagé. Nous avons pu montrer grâce aux méthodes appliquées, que C. *albicans, U. urealyticum, S. agalactiae, G. vaginalis* et C. *non-albicans* ont été les principaux pathogènes responsables des infections cervico-vaginales chez les patientes. Les germes isolés étaient présents chez 51,8 *%* des femmes avec une flore vaginale de type IV. La qPCR a permis de confirmer les absences de *B. crocidurae* et *T. pallidum* chez les patientes. La comparaison de l'examen cytobactériologique et de la qPCR semble indiquer que la qPCR détecte plus de *S. agalactiae,* mais des travaux supplémentaires avec davantage d’échantillons sont nécessaires pour confirmer cette tendance. La qPCR *S. agalactiae* avait une faible sensibilité (40,7 %) et une forte spécificité (94 %). La concordance entre la culture et la qPCR dans le diagnostic de S. *agalactiae* était faible (kappa = 0,33).

Cette étude présente un certain nombre de limites. La collecte des échantillons s'est cantonnée à l'HPD. Même si les patientes proviennent de plusieurs localités dans Dakar ou les autres régions, les données des autres structures hospitalières auraient apporté une plus-value à ce travail. La faiblesse des échantillons positifs à *N.gonorrhoeae* et *C. trachomatis* nous a empêchés de comparer les deux méthodes de diagnostic et surtout d'exposer les avantages de la qPCR dans le diagnostic de ces deux pathogènes. Cette étude a été intégrée dans le fonctionnement courant du laboratoire de bactériologie. De ce fait, pour certains pathogènes, le diagnostic était réalisé suivant les méthodes habituelles, soit par test diagnostic rapide (TDR) pour les pathogènes de culture difficile, soit par culture. Ainsi la comparaison des analyses par TDR et par culture était impossible. À noter également que les qPCR utilisées dans cette étude ne permettent pas de faire la distinction entre un fragment de pathogène et l'agent sous sa forme infectieuse.

Actuellement, la méthode usuelle pour détecter S. *agalactiae* dans les écouvillons cervicovaginaux à l'HPD est la culture sur gélose au sang associée aux TDR antigéniques. Les 19 échantillons positifs à la qPCR et négatifs à la culture semblent indiquer que la qPCR a détecté des pathogènes non viables.

La faiblesse de notre échantillon de travail a entraîné une réduction de la sensibilité de la qPCR. La spécificité de la qPCR était par contre élevée. L'utilisation des milieux d'enrichissement augmenterait la sensibilité de la qPCR ainsi que celle de la culture. La valeur prédictive négative de la qPCR était également élevée, ce qui indique la fiabilité dans le dépistage des échantillons négatifs et qui pourrait contribuer à empêcher le recours inutile à l'antibiothérapie chez les femmes non porteuses de S. *agalactiae.*

Le portage vaginal du S. *agalactiae* chez les femmes enceintes peut être une cause d'infection chez les nouveau-nés. Un dépistage par la qPCR à l'approche de l'accouchement pourrait aider à la prévention des infections chez ces dernières.

Les techniques moléculaires peuvent se révéler intéressantes pour les cliniciens dans le traitement des malades. Dans le moyen terme, l'intégration de la qPCR dans la panoplie de tests utilisés par le laboratoire de l'HPD devrait être envisagée. Elle permettra à ce laboratoire de franchir un palier dans le diagnostic des pathogènes sensibles et difficilement cultivables. Les kits de PCR multiplex (exemple : Allplex™ STI) sont actuellement disponibles et pourraient aider à identifier rapidement les pathogènes en cause. Leur intégration dans un « Point Of Care » serait complémentaire aux examens cytobactériologiques. Mais cela ne doit pas se faire au détriment de la cytobactériologie qui reste indispensable pour aider à mieux évaluer la résistance aux antibiotiques. La collaboration avec les cliniciens devra donc être renforcée dans le but de mettre en place des panels de diagnostic qui contribueraient à améliorer la prise en charge des patients.

## Conclusion

Les laboratoires de microbiologie ont beaucoup évolué ces dernières années. Les nouveaux procédés ont apporté des changements profonds dans la qualité des résultats et la prise en charge des patients. Une fois que les systèmes sont installés, le diagnostic moléculaire peut s'avérer beaucoup plus simple et moins coûteux que l'examen bactériologique. Il pourrait permettre de ramener le coût du diagnostic à 2 624 FCFA (4 €) par patient à moyen terme. Le temps nécessaire à l'identification du pathogène est également beaucoup plus court. Les patients bénéficieront d'une prise en charge précoce, d'un traitement adéquat, d'une réduction des coûts de traitement et d'une baisse de la probabilité de complications. Cependant la culture reste indispensable pour une meilleure étude de la sensibilité aux antibiotiques. Les résultats suggèrent que la qPCR peut être une alternative au moins pour le diagnostic de *S. agalactiae* chez les femmes enceintes avant l'accouchement. Concernant *N. gonorrhoeae* et *C. trachomatis,* des études supplémentaires tenant compte des signes cliniques d'infections par ces germes pour augmenter la probabilité de rencontrer des patients infectés seront nécessaires. Le test Système Amplix qPCR CT/NG/Mycoplasme (C. *trachomatis,* N. *gonorrhoeae, M. genitalium)* est en train dans ce cadre d’être intégré dans le diagnostic de routine des IST à la Fédération des laboratoires de l'HPD.

## Contribution des auteurs

Conception du projet d’étude initial, réalisation des prélèvements, revue de la littérature, rédaction du manuscrit, traitement des données : DIALLO A. S.

Supervision de l’étude : FALL B., SOKHNA C., BASSÈNE H.

Apport critique, correction et approbation de la version finale du document : BASSÈNE H., FALL B., DIEYE Y.

Analyse moléculaire, validation des résultats : DIALLO A. S., SAMBOU M.

Acquisition de financement : SOKHNA C.

Collecte des données générales, analyses et validation des résultats, identification des souches bactériennes : NGOM M., MBACKÉ DAFFE S. M.

## Liens d'intérêt

Les auteurs n'ont aucun conflit d'intérêts à déclarer. Les bailleurs de fonds n'ont joué aucun rôle dans la conception et la réalisation de l’étude. De même, ils n'ont été impliqués ni dans la collecte, la gestion, l'analyse et l'interprétation des données, ni dans la préparation, l'examen ou l'approbation du manuscrit.

## Apport de la qPCR dans le diagnostic des infections cervicovaginales à l'Hôpital Principal de Dakar, Sénégal

### Contribution of qPCR to the diagnosis of cervico-vaginal infections at the Hôpital Principal de Dakar, Senegal

**Tableau III:** Prévalence des germes isolés chez les 346 patientes, Hôpital Principal de Dakar, 2021 Prevalence of germs isolated among 346 patients, Hôpital Principal de Dakar, 2021

SGB		Culture		Total
		(+)	(-)	
**qPCR**	(+)	11	19	30
	(-)	16	300	316
**Total**		27	319	346

Sensibilité : 40,74 % Spécificité : 94,04 % VPP : 36,67 % VPN : 94,94 %

**Tableau III:** Prévalence des germes isolés chez les 346 patientes, Hôpital Principal de Dakar, 2021 Prevalence of germs isolated among 346 patients, Hôpital Principal de Dakar, 2021

*C. trachomatis*		Culture		Total
		(+)	(-)	
**qPCR**	(+)	0	2	2
	(-)	6	142	148
**Total**		6	145	150

Sensibilité : 0 % Spécificité : 98,61 % VPP: 0 % VPN : 95,95 %

**Tableau III:** Prévalence des germes isolés chez les 346 patientes, Hôpital Principal de Dakar, 2021 Prevalence of germs isolated among 346 patients, Hôpital Principal de Dakar, 2021

*N. gonorrhoeae*		Culture		Total
		(+)	(-)	
**qPCR**	(+)	0	3	3
	(-)	1	342	343
**Total**	1	345	346

Sensibilité : 0 % Spécificité : 99,13 % VPP: 0 % VPN : 99,71 %
